# Postbooster Antibodies from Humans as Source of Diphtheria Antitoxin

**DOI:** 10.3201/eid2207.151670

**Published:** 2016-07

**Authors:** Jesús F. Bermejo-Martin, Ana Avila-Alonso, Milagros González-Rivera, Eduardo Tamayo, Jose María Eiros, Raquel Almansa

**Affiliations:** Hospital Clínico Universitario de Valladolid, Valladolid, Spain (J.F. Bermejo-Martin, A. Avila-Alonso, E. Tamayo, J. María Eiros, R. Almansa);; Hospital General Universitario Gregorio Marañón, Madrid, Spain (M. González-Rivera)

**Keywords:** diphtheria, antitoxin, booster, vaccination, antibodies, bacteria, Corynebacterium diphtheriae

## Abstract

Diphtheria antitoxin for therapeutic use is in limited supply. A potential source might be affinity-purified antibodies originally derived from plasma of adults who received a booster dose of a vaccine containing diphtheria toxoid. These antibodies might be useful for treating even severe cases of diphtheria.

Although diphtheria is an almost forgotten disease in industrialized countries, sporadic cases still occur. Possible reasons for these cases include partial failure of vaccine compliance, antivaccine campaigns, inadequate booster regimens, and immunosenescence. Health authority interest in this disease was rekindled after a nonvaccinated boy in Spain died of systemic diphtheria in June 2015 and 9 cases of cutaneous diphtheria among refugees were notified by Denmark, Sweden, and Germany in 2015 (*1*). According to the World Health Organization, 7,321 cases of diphtheria were reported worldwide in 2014. In the early 1890s, Emil von Behring used serum from a hyperimmune horse (challenged with sublethal dose of *Corynebacterium diphtheriae*) to develop equine diphtheria antitoxin (DAT), which seemed to confer passive immunity to patients with diphtheria (*2*). Subsequently, use of equine DAT to treat this disease became common. Uncontrolled but large studies of mortality rates from that time suggested effectiveness of equine DAT use; however, double-blinded randomized studies conducted by Adolf Bingel in 1918 concluded that equine DAT offered no benefit over serum from nonhyperimmune horses (not challenged with *C. diphtheriae*) (*2*). Although modern efficacy studies are lacking, equine DAT is still the recommended treatment for diphtheria, listed among the World Health Organization essential medicines (*3*). When administered early in the clinical course of disease, treatment with DAT can be lifesaving for patients with toxin-induced systemic symptoms.

A large proportion of European countries do not stockpile DAT, and many countries have experienced difficulties replacing expired stockpiles (*3*,*4*). As highlighted by the European Centre for Disease Prevention and Control (*1*), the current lack of DAT in the European Union is a concern. DAT is not produced or licensed in the United States or in most European countries; it is imported from Brazil under an Investigational New Drug protocol (*5*). 

Equine DAT can induce anaphylactic reactions (a test for sensitivity to DAT should be conducted before each administration) (*5*). The European Centre for Disease Prevention and Control and the US Centers for Disease Control and Prevention encourage searching for new providers of equine DAT and promote the development of alternative antitoxins of human origin. The definitive solution will probably come from monoclonal antibodies (*4*) or synthetic molecules such as nucleic acid aptamers. These new molecules could constitute an unlimited source of DAT, with a low risk for hypersensitivity reactions. Unfortunately, these alternatives are not yet available and will need to undergo thorough regulatory processes before being approved for use in humans. We therefore describe the potential role of human plasma from vaccinated volunteers as a source of DAT. 

Plasma from vaccinated persons is used to produce Anthrasil (Cangene Corporation, Winnipeg, Manitoba, Canada), a fully human polyclonal antianthrax intravenous immunoglobulin (IVIG) licensed in the United States. Antitetanus immunoglobulin is produced from plasma of young volunteers who received a booster dose of the tetanus–diphtheria vaccine.

The successful implementation of vaccination programs in industrialized and many developing countries indicates that most of these populations have antibodies against the diphtheria toxin. Nonetheless, the geometric mean concentration of IgG against diphtheria toxin in plasma of vaccinated adults who received the last dose of tetanus–diphtheria vaccine in their adolescence is not much over 0.3 IU/mL (*6*). For diphtheria treatment, 20,000–100,000 IU of DAT is needed; the dose depends on disease severity (*5*). In consequence, producing DAT from plasma obtained from the general population could not be cost-effective because large volumes would be needed to obtain a dose of DAT with enough potency for clinical use. 

An alternative could be to obtain plasma from adult donors who recently received a booster dose of vaccine. Researchers have observed that during the diphtheria epidemic that emerged in the newly independent states of the former Soviet Union from 1991 through 1994, booster vaccination of convalescent patients led to enhanced antidiphtheria toxin titers (*3*,*7*). Seroepidemiologic studies evaluating the effect of booster vaccination of adults against diphtheria support this finding. Booster vaccination of adults induces up to 10 IU/mL of IgG against diphtheria toxin in plasma 4 weeks after vaccination (*8*–*13*) (Table). The use of conjugate vaccines, or a high vaccine dose, could yield the highest plasma concentrations of DAT after a booster dose of vaccine (*9*).

**Table:** Seroepidemiologic studies assessing levels of antidiphtheria antibodies in adults who received a booster dose of vaccine*

Ref.	Study population			Vaccine		Immunogenicity, GMC IU/mL (95% CI)	
	Mean age, y (SD or range)	No.				Before booster	After booster
(*8*)	40.1 (13.63)	1,448		0.5 mL Tdap (Boostrix; GlaxoSmithKline Biologicals, Rixensart, Belgium)		0.4 (0.4–0.4)	4.7 (4.4–5.1)
	40.4 (13.48)	728		0.5 mL Tdap (Adacel; Sanofi Pasteur, Swiftwater, PA, USA)		0.5 (0.4–0.5)	5.0 (4.6–5.4)
(*9*)	31.7 (15–69)	64		0.5 mL of Tdap (Sanofi Pasteur Limited, Toronto, ON, Canada) after previous vaccination with MCV4D (Menactra; Sanofi Pasteur, Swiftwater, PA, USA)		4.45 (2.77–7.15)	8.70 (6.59–11.5)
		379		0.5 mL Tdap (Sanofi Pasteur Limited, Toronto)		0.13 (0.11–0.16)	2.17 (1.84–2.56)
(*10*)	19.4 (1.2)	55		0.2 mL DTap (Kaketsuke, Kumamoto, Japan)		0.22 (0.16–0.30)	4.29 (3.53–5.21)
	19.4 (0.8)	56		0.5 mL DTap (Kaketsuke)		0.21 (0.15–0.30)	6.28 (4.86–8.11)
(*11*)	66.0 (59–91)	252		0.5 mL Tdap (Repevax; Sanofi Pasteur MSD GmbH, Leimen, Germany)		0.04 (0.03–0.06)	1.09 (0.81–1.46)
	24.0 (20–33)	21				0.14 (0.05–0.33)	4.16 (2.36–7.34)
(*12*)	21.1 (0.31)	74		0.5 mL Tdap (Boostrix)		0.3 (0.2–0.4)	6.0 (4.7–7.7)
(*13*)	26.5 (18–52)	401		0.5 mL TdaP (Statens Serum Institut, Copenhagen, Denmark)		0.11 (0.9–0.14)	4.60 (4.03–5.26)
	26.1 (18–55)	399		0.5 mL diTeBooster (Statens Serum Institut)		0.11 (0.09–0.14)	5.54 (4.00–5.15)

*DTaP, diphtheria, tetanus, and pertussis vaccine (for children >6 years of age); GMC, geometric mean concentration; Ref., reference; Tdap, tetanus, diphtheria, and pertussis vaccine (for children >11 years of age and adults).

Assuming use of revaccinated donor plasma with the highest titer, IVIG with a DAT potency up to 60–100 IU/mL could be obtained by using the standard methods for producing IVIG (*3*). This concentration could be enough to treat moderate forms of diphtheria (those with skin lesions only, laryngeal disease, or nasopharyngeal disease) (*5*). The European Pharmacopoeia recommends that the potency of equine-derived DAT be no less than 1,000 IU/mL (*3*). To treat severe diphtheria, a dose of 100,000 IU, obtained by using a 5% IVIG solution with potency of 100 IU/mL, would require 1.6 liters of product, a substantially high volume that would be very difficult to administer to a child. 

This major drawback could be solved by using antigen-specific antibody purification. The process is simple: the antigen is immobilized in a solid phase so that the antibodies that bind specifically to it are retained during addition of plasma. Bound antibody can be recovered by acid elution (*14*). This method has been successfully used to purify specific antibodies from plasma or normal IVIG for research and development purposes (*15*). In 1988, also in an experimental context, M. Sutjita et al. demonstrated that this approach was useful for concentrating DAT from human serum; they used a diphtheria toxoid-Sepharose 4B (Sigma Aldrich, St. Louis, MO, USA) affinity column (*14*). In consequence, this approach could be used to purify DAT from plasma of revaccinated persons or from commercial immunoglobulins (i.e., the antitetanus immunoglobulin itself or nonspecific IVIG), which contains variable concentrations of DAT (Technical Appendix). This concentrated DAT could be useful for treating diphtheria of any severity in adults and children, with very low risk of inducing hypersensitivity reactions.

A potential drawback of affinity purification is that the obtained DAT could be denatured by acid elution. This risk could be minimized by immediately neutralizing pH by adding 1 mol/L Tris, followed by dialysis with phosphate-buffered saline. The obtained product should undergo the same biological agent removal processes as those used for standard IVIG (i.e., chemical inactivation, heat inactivation, nanofiltration, and precipitations). Neutralization potency of DAT obtained from human plasma should be assigned according to the Vero cell cytotoxicity assay and the guinea pig lethality model; the 1st International Standard for Diphtheria Antitoxin Human should be used as the reference antitoxin (National Institute for Biological Standards and Control code 10/262).

A limitation of using DAT obtained from human plasma is the potential cost. Some developing countries, where most cases of diphtheria occur, could not afford it. Production costs and the price of each dose of human DAT could be reduced by using as source the same plasma obtained from the donors recruited to produce the antitetanus immunoglobulin. Industrialized countries could also donate doses of this human DAT to developing countries.

Plasma from young adults receiving a booster dose of vaccine could represent a potential source of human DAT. Antigen-affinity antibody purification could help to produce a highly concentrated DAT from this plasma, useful for treating even the most severe forms of diphtheria. This approach could help mitigate the limited access to this essential medicine.

Technical Appendix Concentration of diphtheria antitoxin in commercially available intravenous immunoglobulins.
