# Supplementary material for: Postbooster Antibodies from Humans as Source of Diphtheria Antitoxin
Source: Emerg Infect Dis. 2016 Jul;22(7):1265–7. doi: 10.3201/eid2207.151670 (PMC4918160; doi:10.3201/eid2207.151670)
Supplement: Technical Appendix — Concentration of diphtheria antitoxin in commercially available intravenous immunoglobulins. [file 15-1670-Techapp-s1.pdf]

Article DOI: http://dx.doi.org/10.3201/eid2207.151670

Postbooster Antibodies from Humans as Source of Diphtheria Antitoxin

Technical Appendix

Technical Appendix Table. Content in diphtheria antitoxin in commercially available intravenous immunoglobulins

Intravenous immunoglobulin	Antidiphtheria IgG, IU/mL, mean, (SD or range)	No. lots evaluated	Method for evaluation of diphtheria antitoxin	Reference
Endobulin (Baxter)	7.82 (5.2)	3	In-house ELISA with standard calibrated against “Diphtheria antitoxin human serum 91/534” NIBSC reagent	(*1*)
Flebogamma 5% (Grifols)	19.9 (19.3)	6		
Octagam (Octapharma)	10.1 (6.5)	16		
Tegeline (LFB-Biomedicaments)	7.8 (4.1)	9		
Vigam (Bio Products Laboratory)	8.9 (5.4)	2		
Immunoglobulin (NA)	12.9 (6.9)	2		
BivIgam (Biotest)	18.2 (3.5)	NA	NA	(*2*)
TBSF (Taiwan Blood Services Foundation)	4.46 (0.86)	8	ELISA from Virotech® (Genzyme/Sekisui®)	(*3*)
Carimune (CSL Behring)	3.6 (NA)	NA	Diphtheria toxin neutralization assay	(*4*)
Flebogamma 10% DIF (Grifols)	13.7 (1.4)	NA	NA	
Gammagard S/D 5% (Baxter)	5 (NA)	NA	Diphtheria toxin neutralization assay	
Gammaplex 5% (Bio Products Laboratory)	2.2 (NA)	NA	NA	
Hizentra 20% (CSL Behring)	2.5 (NA)	NA	NA	
Octagam 5% (Octapharma)	5-30	NA	NA	
PrivIgen 10% (CSL Behring)	4.9 (3.8-7.3)	NA	NA	
Flebogamma 5% DIF (Grifols)	6.0 (1)	29	ELISA (manufacturer not provided)	(*5*)
NA (CSL Behring)	3.6 (1.1)	44	ELISA from Scimedx®	(*6*)

*NA, not available.

References

1. Nobre FA, Gonzalez IG, Simão RM, de Moraes Pinto MI, Costa-Carvalho BT. Antibody levels to tetanus, diphtheria, measles and varicella in patients with primary immunodeficiency undergoing intravenous immunoglobulin therapy: a prospective study. BMC Immunol. 2014;15:26. PubMed http://dx.doi.org/10.1186/1471-2172-15-26

2. Wasserman RL. A new intravenous immunoglobulin (BIVIGAM®) for primary humoral immunodeficiency. Expert Rev Clin Immunol. 2014;10:325–37. PubMed http://dx.doi.org/10.1586/1744666X.2014.891438

3. Wu C-Y, Wang H-C, Wang K-T, Yang-Chih Shih D, Lo C-F, Wang D-Y. Analyzing titers of antibodies against bacterial and viral antigens, and bacterial toxoids in the intravenous immunoglobulins utilized in Taiwan. Biologicals. 2013;41:88–92. PubMed

4. Siegel J. Immune globulins: therapeutic, pharmaceutical, cost, and administration considerations. Pharm Pract News [cited 2016 Apr 27]. http://www.pharmacypracticenews.com/download/PF131_ImmuneGlobulins_WM.pdf

5. Jorquera JI. Flebogamma 5% DIF development: rationale for a new option in intravenous immunoglobulin therapy. Clin Exp Immunol. 2009;157(Suppl 1):17–21. PubMed http://dx.doi.org/10.1111/j.1365-2249.2009.03953.x

6. Lejtenyi D, Mazer B. Consistency of protective antibody levels across lots of intravenous immunoglobulin preparations. J Allergy Clin Immunol. 2008;121:254–5. PubMed http://dx.doi.org/10.1016/j.jaci.2007.11.001
